# Biospeckle optical coherence tomography in speedy visualizing effects of foliar application of plant growth hormone to Chinese chives leaves

**DOI:** 10.1186/s13104-020-05219-7

**Published:** 2020-08-08

**Authors:** Uma Maheswari Rajagopalan, Mahjabin Kabir, Yiheng Lim, Hirofumi Kadono

**Affiliations:** 1 Department of Mechanical Engineering, Shibaura Institute of Technology, 3-7-5 Toyosu, Koto City, Tokyo 135-8548 Japan; 2 Graduate School of Science and Engineering, Saitama University, 255 Shimo okubo, Sakura-ku, Saitama, 338-8570 Japan; 3 Department of Farm Power and Machinery, Bangladesh Agricultural University, Mymensingh, 2202 Bangladesh

**Keywords:** Biospeckle, Optical coherence tomography, Chinese chives, Biospeckles, Phytohormone, Gibberellic acid (GA_3_), Speckle contrast, Structural image, Leaf surface, Plant physiology

## Abstract

**Objective:**

The aim of this study is to demonstrate the potential of applying the contrast of the speckles obtained as noise in optical coherence tomography (OCT) images to monitor short term activity changes during foliar application of phytohormones to a plant leaf. Plant growth hormone, gibberellic acid (GA_3_) was sprayed onto the leaf of Chinese chives and after 60 min, OCT images (1 frame: 512 × 2048 pixels) were recorded at ten frames per second for a few tens of seconds.

**Results:**

Contrast across the temporal axis was calculated for each pixel of the structural images and biospeckle OCT contrast images were obtained under the conditions of before and after application of GA_3_ for different concentrations 0, 40, and 100 μM. Application of 40 μM GA_3_ failed to show any differences in the OCT structural images. However, bOCT contrast image was clearly different. Changes were found to be statistically significant. Although the mechanism for the contrast difference is not clear, it can be said there is a large change across the temporal scale with the application of GA_3_. Demonstration of OCT utilizing the speckle contrast is believed to have the potential as a promising tool in plant physiology.

## Introduction

Optical coherence tomography (OCT) maps in three dimensions of a structure by utilizing the inherent reflectivity variations arising as a result of optical refractive index variations within a biological tissue [7, 8]. OCT is a non-contact and non-destructive technique providing in vivo tomographic images of the internal tissue structure and is widely used in the biomedical fields of ophthalmology, dermatology [6] and also endoscopic applications of angiography.

However, the potential of OCT in the field of plant biology and botany has still been under investigated. Most recently, OCT has been used in in vitro study of autumnal color change of maple leaves and their associated changes in optical properties [3]. There have been reports of visualizing the structural properties of apple peel [13], pathogen attack of orchids [5], and capsicum seed germination studies [14].

It should be pointed out that the potential of OCT in monitoring structural changes of plants in relation to environmental variations has been very limited. Our group earlier reported the use of monitoring changes in the activity with respect to Ozone exposure of rice plants [11, 12] and also more recently with changes in the germination patterns of seeds with environmental changes [10] thus demonstrating the potential of not just structure visualizations but also capability of OCT in visualizing environment induced changes in plants.

In such monitoring, we proposed the use of laser speckles that have a granular appearance and are, in general, considered to be a noise degrading the structural images. The speckles invariably arise from the random interference of the scattered light when an optically rough surface or a scattering medium object is subject to the illumination of a coherent light source such as a laser source. If the object is stationary with no motion of the surface structure of the object, the intensity of the speckle patterns remain temporally stable. However, when the object is dynamic, i.e., there is a continuous movement of the scattering centers as in the case of biological samples, the speckle pattern varies, and there is a fluctuation in the intensity. This dynamic speckle pattern is a specific characteristic of the biological tissues and thus has been named as biospeckle and could be used to characterize the activity of the biological sample [1, 2, 16]. The origin of the biospeckles is not yet clear and is considered to be due to biological processes such as cytoplasmic streaming, organelle movement, cell growth and division, and biochemical reactions occurring within the plant. Such activities are expected to be changed under environmental changes.

In this study, the temporal variation of each pixel of the structural images or OCT reflectivity signals were characterized by a parameter called biospeckle contrast which is defined as the ratio of the standard deviation to the mean intensity of a pixel calculated over a few tens of seconds. The study was done before and following application of a phytohormone, gibberellic acid (GA_3_), after an hour.

GA_3_ is considered to be an important phytohormone responsible for green revolution and is widely used either on its own or with other growth hormones such as auxin or cytokinin to enhance the growth [4, 15]. Thus, techniques that require monitoring the action of plant hormones within an intact tissue providing high temporal and spatial resolutions are needed. Leaf of *Allium tuberosum*, commonly known as Chinese chives, was exposed to different concentrations of GA_3_, and OCT structural images were recorded to calculate the biospeckle contrast images. Fairly high concentration of GA_3_ was first tested for the changes in contrast, and then nominal concentrations were used. Results revealed that nominal concentrations could show difference in contrast before and after the application of GA3 while structural images could not.

## Main text

### Experimental system of Spectral Domain Optical Coherence Tomography (SD-OCT)

**Fig. 1:**
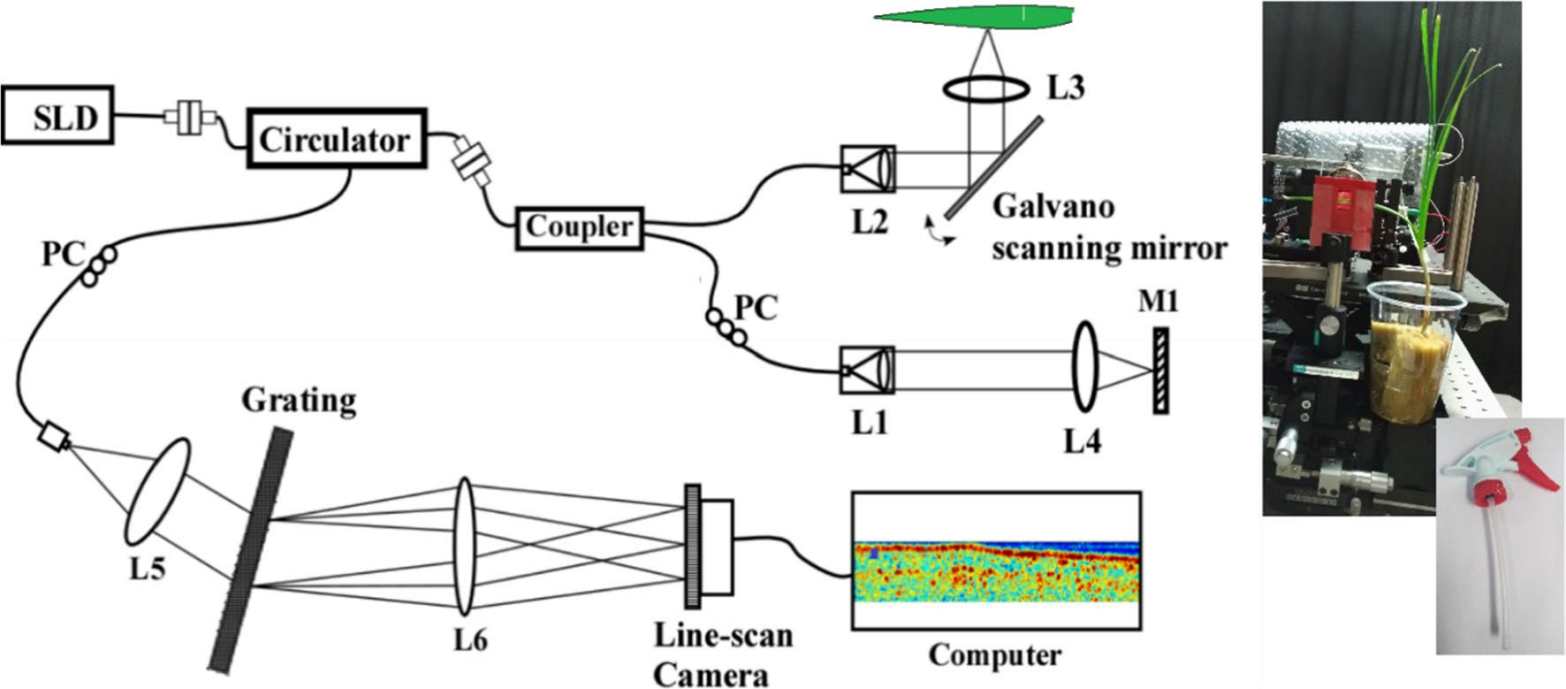
A schematic of the experimental biospeckle Optical Coherence Tomography system with an inset of the leaf mounted for scanning and the spray used for foliar application of the phytohormone GA_3_. *SLD* super luminescent diode, *L1–L6* lenses used in the system. NA of objective lens L4 is 0.024. Here, the incident power delivered to the leaf sample was approximately 5 mW, and it was made sure that such an irradiance was below the damage threshold for the plant leaves

Healthy young leaves of Chinese chives grown from seeds, 2 weeks old, were used as samples (details on growing conditions given in Additional file 1). A schematic diagram of the SD-OCT system constructed using optical fibers is shown in Fig. 1. The light source used in the system is a super luminescent diode (SUPERLUM, SLD-371-HP3-DBUT-SM-PD, Ireland) with a central wavelength 836.1 nm and a bandwidth of 55.2 nm providing a total outpower of 15.6 mW. Light from the source coupled first to the input port of a circulator (AC Photonics, Inc. USA) is further divided into two beams to illuminate, respectively, the sample and reference arms by a 2 × 2 50/50 fiber coupler (TW850R5A2—2 × 2 Wideband Fiber Optic Coupler, 850 ± 100 nm, THORLABS, UK). The axial resolution (depth resolution) of the system is given by,

1 $$ \Delta z = \frac{{2\ln \lambda_{o}^{2} }}{\pi n\Delta \lambda }, $$

where λ_o_ and ∆λ are the central wavelength and band width of the light source, respectively. n is the refractive index of the tissue and assumed to be 1.4. The depth resolution in free space was estimated to be 6 μm in air and the lateral resolution was estimated to be 22 μm.

Reflected light beams from the reference mirror and the sample arms are again combined at the same coupler to be passed through the circulator. The reference arm consists of a collimating lens L1, objective lens L4, and mirror M1. On the other hand, the sample arm contains a collimating lens L2 to illuminate Galvano mirrors (Model_GVS012, THORLABS, UK) by which the light beam is scanned laterally in the x and y directions to be focused through an imaging lens L3 (LSM03-BB—Scan Lens, EFL = 36 mm, THORLABS, UK) across the sample to generate 3D OCT structural images. The light from the output port of the circulator is collimated by a lens L5 to pass through a custom-built spectrometer consisting of a grating, a lens L6 (focal length, 200 mm) and a line-scan camera (L104k-2 k, BASLER, Germany). The spectral interference signal was first converted from the wavelength space to a wavenumber space through rescaling and then Fourier transformed to obtain the depth resolved reflectivity profile of the sample. Lateral scanning was done by scanning the galvano-mirror acquiring x–z scan images. One z-scan was acquired in 250 μsec and x–z image frames were acquired at an acquisition rate of 10 frames per second. Image size was fixed to be 2048 × 512 pixels.

Biospeckle contrast, C, was defined as the ratio of the standard deviation of the intensity at each pixel along time to the mean value of the pixel across the total time of scans. When speckle contrast is large, it could mean a large variation from mean across the frames and thus large temporal fluctuations. Presence of large temporal fluctuations in the intensity would indicate for stronger movements within the leaf. Therefore, the magnitude of changes in speckle contrast could be used to predict the dynamics within the leaf.

## Results and discussions

### OCT structural images

**Fig. 2:**
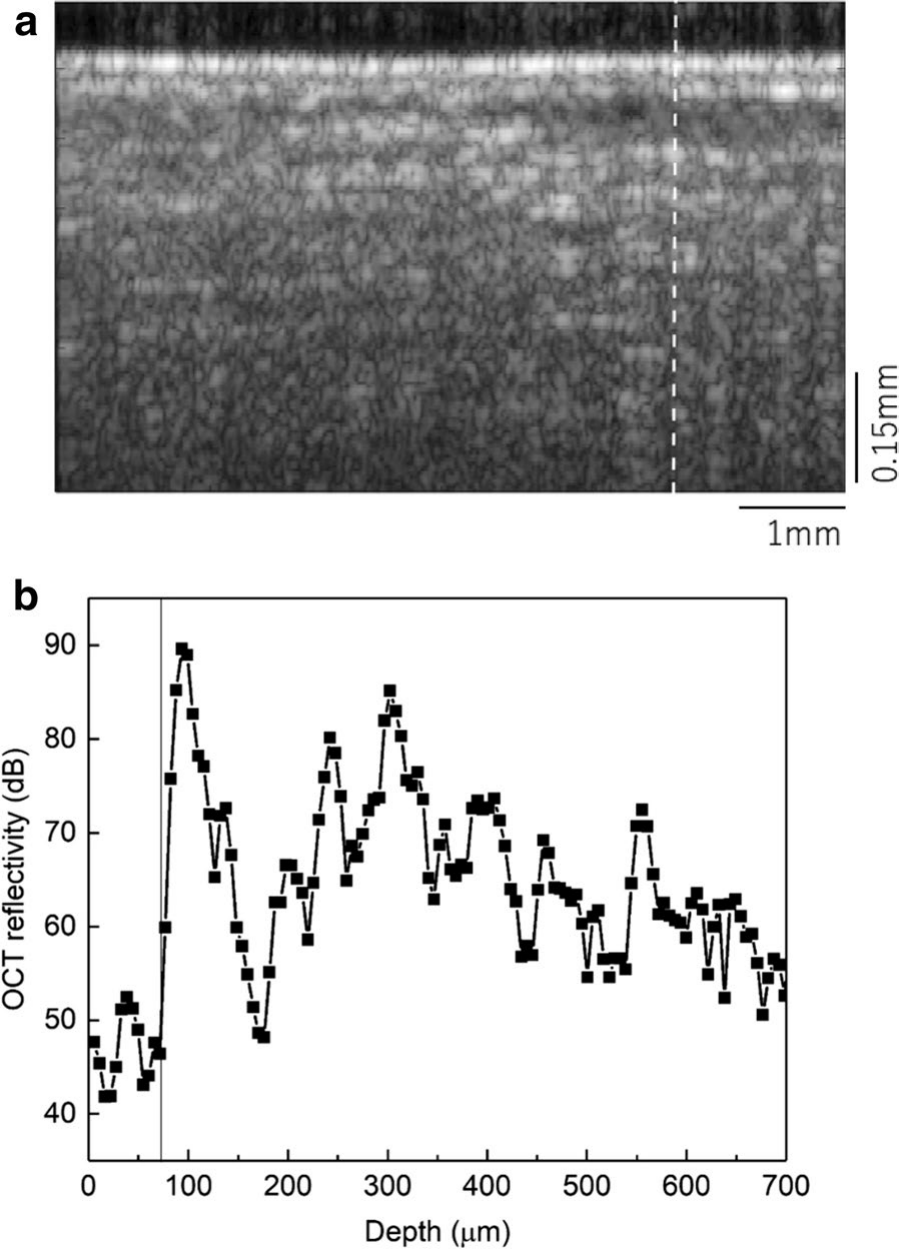
A x–z scan of the averaged OCT reflectivity image, **a** with the intensity depth profile, **b** across the white line indicated in the image. As seen, the intensity variation corresponding to the laminar organization could be seen. Here the averaging is done over 600 frames

Figure 2 shows the averaged OCT image of the Chinese chives leaf before the application of any plant growth hormone, GA_3_. OCT x–z image reflectivity signals can visualize different laminar structure of the internal structure of the leaf with the intensity scale given in log scale (Fig. 2a). Bright regions correspond to stronger OCT reflectivity signal while dark regions correspond to reduced reflectivity. At the very top, i.e., at the air-leaf interface, epidermal layer could be seen with larger reflectivity. Within the tissue, there is a laminar organization of the leaf structure that could be visualized as seen from the reflectivity profile in Fig. 2b also. Because of the inhomogeneous nature of the leaf structure as well as the strong scattering within the tissue, there is noise from scattering with the tissue. Below the mesophyll layer, spongy mesophyll layer and the other layers could be recognized from comparison with anatomical section given in the Additional file 1: Figure S1. Due to stronger scattering within the tissue, there is strong appearance of the granular structure patterns called speckles having strong spatial contrast.

The speckles obtained in the OCT images have been used as a possible method to investigate the internal response due to application of external agent. In general, for a biological tissue like a plant leaf, the incident light undergoes multiple scattering. Change in scattering could happen from change in size, number of scatterers or both. In our case, we expect application of GA3 introduces structural variations to be reflected as bOCT contrast changes.

### Biospeckle contrast results

**Fig. 3:**
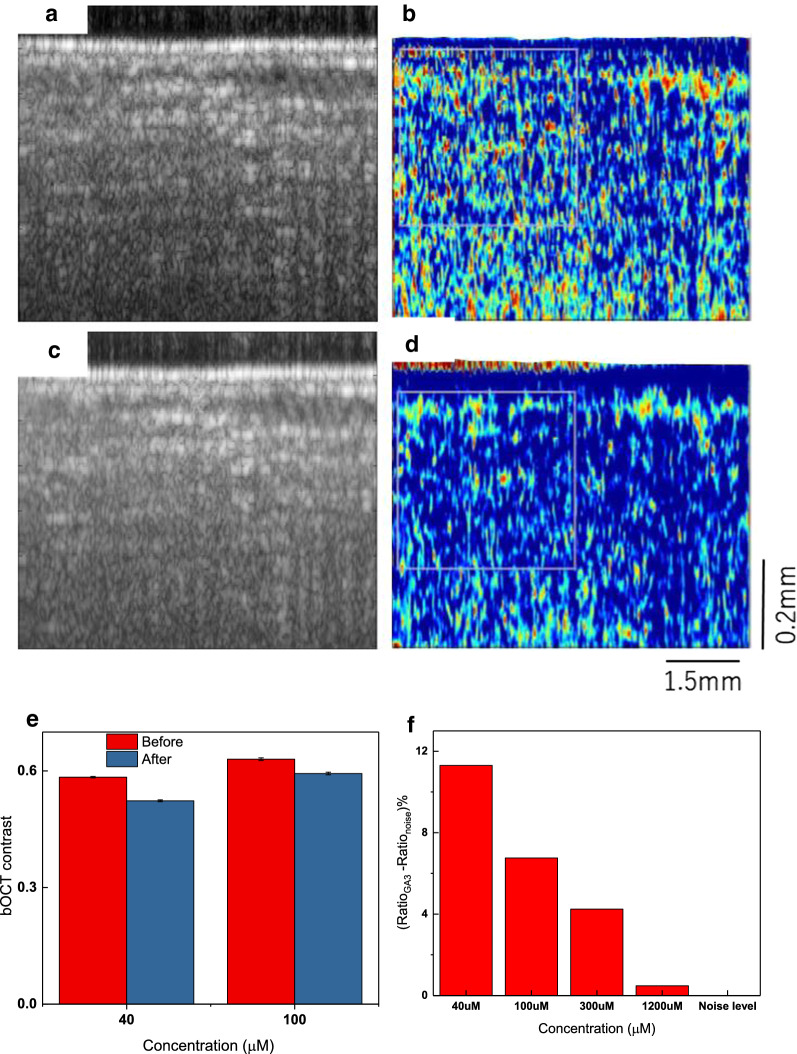
Averaged OCT structural (**a**, **c**) and bOCT contrast (**b**, **d**) images obtained before and after 60 min following the foliar application of 40 μM concentration of phytohormone GA_3_. Averaged bOCT contrast shown in **e** along with the R_diff_ defined as the difference in the ratio for GA3 to the ratio at noise level in % (**f**) (refer for details to Additional file 1)

The conventional OCT cross-sectional imaging (Fig. 2a), could visualize internal laminar organization of the Chinese chives leaf structure. In order to validate whether OCT is capable of visualizing changes with foliar application of growth hormone, OCT images had been acquired before and 60 min following application of 40 μM GA_3_. The results of OCT reflectivity structural images are shown, respectively, in Fig. 3a, c. There are no clearly distinguishable changes in the structural images following application of the hormone. This is expected as the visible structural changes to be minimal. However, application of the hormone would change in the internal mobility of the organelle structures, and this is expected to be reflected in the dynamical characteristics of the OCT signal. bOCT images obtained before (Fig. 3b) and after 60 min (Fig. 3d) following the application of 40 μM show a clear difference in the biospeckle contrast which is not seen in the structural images.

When we apply an external agent, such as GA_3_ in this case, this would directly affect the structural organization for example damaging the cellulose microfibril arrangement. There could be loosening of the arrangement leading to possible break down of the arrangement. We hypothesize such changes in arrangement could cause change in back scattering to cause change in scattering, thus speckle intensity.

With the application of the plant growth hormone, there are activity changes within the structure within an hour. Similarly, application of 100 μM GA_3_ resulted in reduction of activity close to the surface as could be identified from Additional file 1: Figures S2b, d in comparison to the structural images (Additional file 1: Figures S2a, c). Further, to confirm for the changes, a relatively higher concentration of 1.2 mM was attempted and the effects in activity reduction could be clearly visualized (Additional file 1: Figure S3) agreeing with our hypothesis. At smaller concentrations, there are still changes in the surface structure which appeared in this case within 60 min or longer.

### Quantitative analysis

To conduct quantitative analysis (Additional file 1 for details), we paid attention to specific local areas, i.e., region of interest (ROI) and calculated the average local contrast of the bOCT images. For each of the bOCT image, six ROIs were selected from the surface and deeper regions as indicated by the rectangles in Additional file 1: Figure S4 (left). Mean local contrast within each of the six ROIs was calculated (Additional file 1: Figure S4 (right)). Averaging was done over ROIs and over the sets of images from the leaf of the same plant and referred to grand average. This was repeated for the conditions of before and after foliar application of GA3 for different concentrations of 40 and 100 μM. t-test was used for verifying the significance of the results between the GA3 exposure conditions and it was found that the results were found to be significant within the confidence level of 95%.

Results of R_diff_ defined to quantitatively assess the effect of the exposure conditions of different concentrations including higher concentrations of 300 μM, 1200 μM and the noise level is shown in Fig. 3f. Noise level corresponded to the null reflectivity signal indicating the variation within 60 min.

$$ {\text{R}}_{\text{diff}} = {\text{ R}}_{\text{GA3}} - {\text{R}}_{\text{noise}} $$

where

$$ {\text{R }} = \, \left( {\langle {\text{C}}\rangle_{\text{before}} - \langle {\text{C}}\rangle_{\text{after}} } \right)/\langle {\text{C}}\rangle_{\text{before}} \times { 1}00 $$

Here R was defined as the difference in grand average bOCT contrast obtained before and after 60 min divided by that obtained before and expressed as a percentage. Results at different concentrations revealed 40 μM to have significant effect agreeing with our earlier results [9]. At higher GA3 concentrations, the layered structure got affected significantly corresponding to dramatic reduction in the temporal changes and thus in the activity within the leaf. Our quantitative results show the potential of bOCT demonstrating wider implications in investigating immediate responses not to just the phytohormone but to harmful external agents too.

## Limitations

Our results suggest bOCT could be used to in in vivo real short time monitoring of Chinese chives leaf under growth hormone application GA_3_. However, the origin of such signals is unclear and is believed to be the result of the dynamical changes happening within the leaf. (2081 words)

## Supplementary information

**Additional file.** Supplemetary details about the plant growing conditions, microtome observations and quantitative analysis along with Figures S1 to S4 are given.

## Data Availability

Data available on request from the author.

## Abbreviations

GA_3_: Gibberellic acid
OCT: Optical coherence tomography
bOCT: Biospeckle optical coherence tomography
SD-OCT: Spectral Domain Optical Coherence Tomography
