# Supplementary material for: Biospeckle optical coherence tomography in speedy visualizing effects of foliar application of plant growth hormone to Chinese chives leaves
Source: BMC Res Notes. 2020 Aug 8;13:377. doi: 10.1186/s13104-020-05219-7 (PMC7414531; doi:10.1186/s13104-020-05219-7)
Supplement: Supplementary file 1 — Additional file. Supplemetary details about the plant growing conditions, microtome observations and quantitative analysis along with Figures S1 to S4 are given. [file 13104_2020_5219_MOESM1_ESM.docx]

**Additional materials**

**Biospeckle optical coherence tomography in visualizing effects of foliar application of plant growth hormone to Chinese chives leaves**

Uma Maheswari Rajagopalan^a^, Mahjabin Kabir^b,c^, Yiheng Lim^b^, Hirofumi Kadono^b,^*

*Plant materials*

Healthy young leaves of Chinese chives grown from seeds, two weeks old, were used as samples. The plants were grown from seeds with the seeds being purchased from a commercial gardening store. Plants were grown in a growth chamber (Conviron, Controlled Environmental Ltd, Winnipeg, Manitoba, Canada). The conditions of the chamber were kept following a day/night cycle of 12h/ 12h at an air temperature of 25^o^C/ 20^o^C, a light intensity of 260 -350 μmolm^-2^s^-1^/0 μmolm^-2^s^-1^, and relative humidity of 55%-65%. Three halogen lamps with the fiber optic light guides (PHL-150, MEJIRO PRECISION, Japan) were used to illuminate the plant with a light intensity of 500 μmolm^-2^s^-1^. The plants were regularly watered. As the plants used were wild with wide usage in Chinese culinary, there were no special restrictions on its usage in the experiments and limitations from the university ethical committee.

Effects of different concentrations of GA_3_ namely, 0, 40 µM, and 100µM were used. As a way, to introduce and confirm the effects of the spray, fairly large concentration of 1200 µM was attempted at first. Foliar pray was done with a piston on to the leaf surface from a distance of around 20 cm so as to wet the leaf surface completely confirming dipping of drops from the surface. A total of three leaves from three different samples were used and either the first latest or the second latest leaf was used in the experiments.

**Microtome observations**

In order to compare the structural observations done by OCT, microtome (MTH-1, J17013, NK system, Nippon Medical & Chemical Instruments Co., Ltd., Osaka, Japan) was used to obtain cross-sections of the leaf. The leaf was sandwiched within a carrot following making a cross and microtomed to slices of nearly 100 μm thick. The slices were immediately observed under an optical microscope to obtain the cross sections. Although the current resolution of the microtome is much lower than that of OCT, it could still provide a cross-sectional view of the internal structure that could be used to compare the anatomical structure.

Statistical analysis

In order to do the statistical analysis, ROIs of the averaged structural scan image was used, and the local mean over the ROI of the bOCT signal was used for evaluation.

At first, a biospeckle image (bOCT) was calculated over hundred scans corresponding to a single set, and the bOCT calculation was repeated for three sets obtained from the same position of the leaf. Next, this process was repeated for two different leaves from three different plants.

For each of the biospeckle images, six ROIs were selected from the surface and deeper regions of the images as indicated by the rectangles in Figure S4 (left). Mean bOCT signal within each of the six ROIs was calculated and shown Figure S4 (right). Here the averaging was done over each of six ROIs and over the sets of images from the leaf of the same plant. This was repeated for the conditions of before and after foliar application of GA_3_ for different concentrations of 40 and 100 μM. t-test was used for testing the significance of the results between the exposure conditions to GA_3_, and it was found that the results were significant within the confidence level of 95%.

**
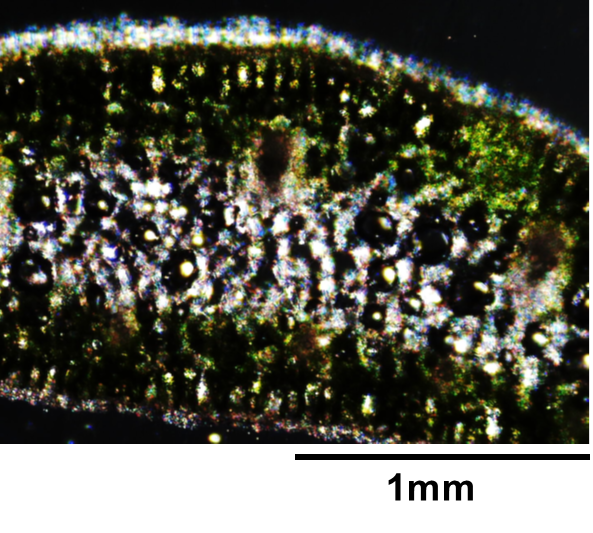
**

**Figure S1.** A microtome image of the laminar organization of the Chinese chive leaf. The thickness of the slice was around 100 μm and is not exactly same as the OCT in vivo slice images.

**(c)**

**(b)**

**(a)**

**(d)**

**Figure S2.** Averaged OCT structural (a,c) and bOCT speckle contrast (b,d) images obtained before and after 60 min following the foliar application of 100 μM concentration of phytohormone GA_3_. Here the averaging is done over six hundred frames. A clear reduction in biospeckle contrast could be observed in the bOCT speckle contrast image while in the structural image, the changes are difficult to distinguish.

**(b)**

**(d)**

**(a)**

**(c)**

**Figure S3.** OCT structural (a,c) and bOCT speckle contrast (b,d) images obtained before and after 120 min following the foliar application of 1200 µM concentration of phytohormone GA_3_. The large concentration was used to change in the activity with the application of GA_3_ and loss in laminar organization could be observed both in the structural as well as the biospeckle contrast images.


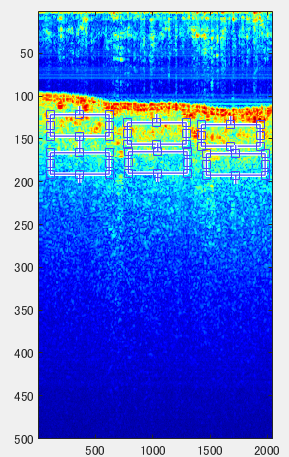


**Figure S4** (left) shows the average OCT structural image obtained by averaging over 100 OCT (x-z) scans with six regions of interest (ROI) indicated by rectangles. Each rectangle corresponds to 512 x 25 pixels. The bOCT speckle contrast for a set calculated from hundred scans was averaged over three sets and this was further averaged over the results obtained for two different leaves from three different plants. Either the latest or the second latest leaves were used in the experiments. Here the red and blue bars represent respectively, the averaged bOCT speckle contrast obtained before and after the foliar application of 40 μM GA_3_.
